# Pheochromocytoma-Induced Leukocytosis With Concurrent Severe Eosinophilia: A Case Report

**DOI:** 10.7759/cureus.98160

**Published:** 2025-11-30

**Authors:** Nouf Alnaqeeb, Mohamed M Abd Elhamid, Ihab AImagdub, Randa Ali

**Affiliations:** 1 Internal Medicine Department, Sheikh Khalifa Medical City, Abu Dhabi, ARE

**Keywords:** eosinophilia, leucocytosis, metanephrines, pheochromocytoma, secondary hypertension, uncontrolled hypertension

## Abstract

Pheochromocytoma is a rare catecholamine-secreting tumor arising from the adrenal medulla. Despite how rare the tumor is, early recognition is critical due to its potential to cause life-threatening complications. Symptoms include episodic hypertension, headaches, and palpitations. Unusual laboratory findings, such as leukocytosis and eosinophilia, are rarely associated with this condition. We report the case of a 42-year-old female presenting with intermittent headaches, episodic hypertension, palpitations, and other symptoms. Initial laboratory investigations showed significant leukocytosis (19.8 x10⁹/L), thrombocytosis (735 x10⁹/L), and eosinophilia (2.7 x10⁹/L), which later reached 5.10 x10⁹/L. Elevated plasma and urinary metanephrines confirmed the diagnosis of pheochromocytoma. CT abdomen showed a large right adrenal mass (10.2 × 9.4 × 9.0 cm) with cystic and hemorrhagic features. Workup to exclude infectious, allergic, and myeloproliferative causes of eosinophilia and leukocytosis was unremarkable. The patient was stabilized medically with alpha- and beta-blockers and scheduled for preoperative embolization followed by laparoscopic adrenalectomy. Pheochromocytoma may present with atypical hematologic abnormalities such as eosinophilia and leukocytosis. This case highlights a rare association between pheochromocytoma and both eosinophilia and leukocytosis. Possible mechanisms include tumor-related secretion of pro-inflammatory cytokines like IL-6 or catecholamine-mediated bone marrow stimulation. Recognition of such atypical presentations can prevent diagnostic delays and facilitate early intervention. This case also highlights an unusual radiological appearance of pheochromocytoma, a large, cystic adrenal mass with internal hemorrhagic changes in a patient with uncontrolled hypertension. Clinicians should consider the diagnosis of phaeochromocytoma in patients with unexplained secondary hypertension and abnormal white cell counts, in the absence of infection or neoplastic disease.

## Introduction

Pheochromocytoma is a rare catecholamine-secreting neuroendocrine tumor that arises from the chromaffin cells of the adrenal medulla. It represents an uncommon but important cause of secondary hypertension. The clinical presentation of Pheochromocytoma varies; the most common presenting symptoms are the triad of episodic headache, palpitations, and profuse sweating. These symptoms are often associated with paroxysmal hypertension; however, patients may present with hypertension that is resistant to treatment [1].

Despite its rarity, recognizing pheochromocytoma is important because it can lead to severe complications if left untreated. Early diagnosis and appropriate management are essential to prevent life-threatening events associated with this condition, including bleeding, shock, and death [1].

Pheochromocytoma accounts for approximately 0.2-0.6% of patients with hypertension and represents about 5-7% of adrenal incidentalomas [2]. There is no clear sex predominance. The risk of recurrence after curative surgery has been estimated in a recent systematic review to be approximately 5% at 5 years of follow-up, although single-center series continue to report cumulative recurrence rates ranging from 6.5% to 16.5% [3,4]. Given this ongoing risk, current guidelines recommend long-term annual biochemical surveillance to monitor for recurrence or metastatic disease [4].

Importantly, beyond the classic adrenergic features, growing evidence links pheochromocytoma to systemic effects mediated by catecholamines and other tumour-derived factors, including inflammation, leukocytosis, changes in white cell counts, and acute phase reactants. However, the association of concurrent leukocytosis and severe eosinophilia in a patient with pheochromocytoma, to our knowledge, is not well-documented in the literature [5].

In this case report, we describe a patient with pheochromocytoma presenting with leukocytosis and a potentially rare association with severe eosinophilia.

## Case presentation

A 42-year-old female presented to the emergency department in December 2024 with a history of severe headache for 2 weeks. It started six months before her presentation to the Emergency Department. She described the headaches as intermittent, fluctuating in severity and duration, and worsening over the past two weeks. The headache was located in the frontal area and radiated to the occipital region. Associated with profuse sweating, palpitations, difficulty in sleeping, tremor, and dizziness. These episodes usually lasted for 30 minutes, and they were not relieved by pain medication. She denied any fever, hot flushes, visual changes, diplopia, photophobia, or neurological abnormalities. She also denied chest pain, shortness of breath, weight loss, nausea and vomiting, or any gastrointestinal symptoms. She denied joint pain, stiffness, rash, or mouth ulcers. The patient's past medical history was significant for taking amlodipine/telmisartan 5 mg/40 mg daily for hypertension for 4 years. She reported compliance with the antihypertensives; however, they didn't improve her symptoms most of the time. She denied using any other medications, including oral contraceptive pills (OCPs).

Upon presentation to the emergency department, she was afebrile, her heart rate was 96 bpm, and she had a blood pressure of 156/132 mmHg. Cardiac exam revealed normal S1 and S2 heart sounds, with no added sounds or murmurs. She was euthyroid clinically. A brief neurological exam was unremarkable. Her abdomen was soft, non-tender, with no organomegaly, normal bowel sounds, and no stigmata of Cushing's disease. Laboratory studies were remarkable for leukocytosis, thrombocytosis, and severe eosinophilia (Table 1).

**Table 1:** Serum kidney function, electrolytes, CRP, and hematological tests

Test	On admission	On discharge	Reference range
Na	136 mmol/L	137 mmol/L	135-145 mmol/L
K	5.1 mmol/L	4.4 mmol/L	3.4-5.0 mmol/L
Creatinine	64 umol/L	40 umol/L	62-106 umol/L
eGFR	103	124	>90
CRP	3.3 mg/L	4.4 mg/L	>8 mg/L
WBC	19.8 x10^9^/L	24.4 x10⁹/L	4.5-11 x10⁹/L
RBC	5.37 x10^12^/L	4.36 x10^12^/L	3.9-5.2 x10^12^/L
Hemoglobin	159 g/L	127 g/L	161-150 g/L
Hematocrit	0.47 L/L	0.37 L/L	0.36-0.46 L/L
Platelets	735 x10⁹/L	572 x10⁹/L	140-400 x10⁹/L
Neutrophils	12.33 x10⁹/L	14.29 x10⁹/L	1.8-7.7 x10⁹/L
Lymphocytes	6.15 x10⁹/L	4.10 x10⁹/L	1.0-4.8 x10⁹/L
Monocyte	0.88 x10⁹/L	0.76 x10⁹/L	0.1-0.9 x10⁹/L
Eosinophils	2.70 x10⁹/L	5.10 xx10⁹/L	0-0.5 x10⁹/L
Basophils	0.16 x10⁹/L	0.12 x10⁹/L	0-0.1 x10⁹/L

**Figure 1:**
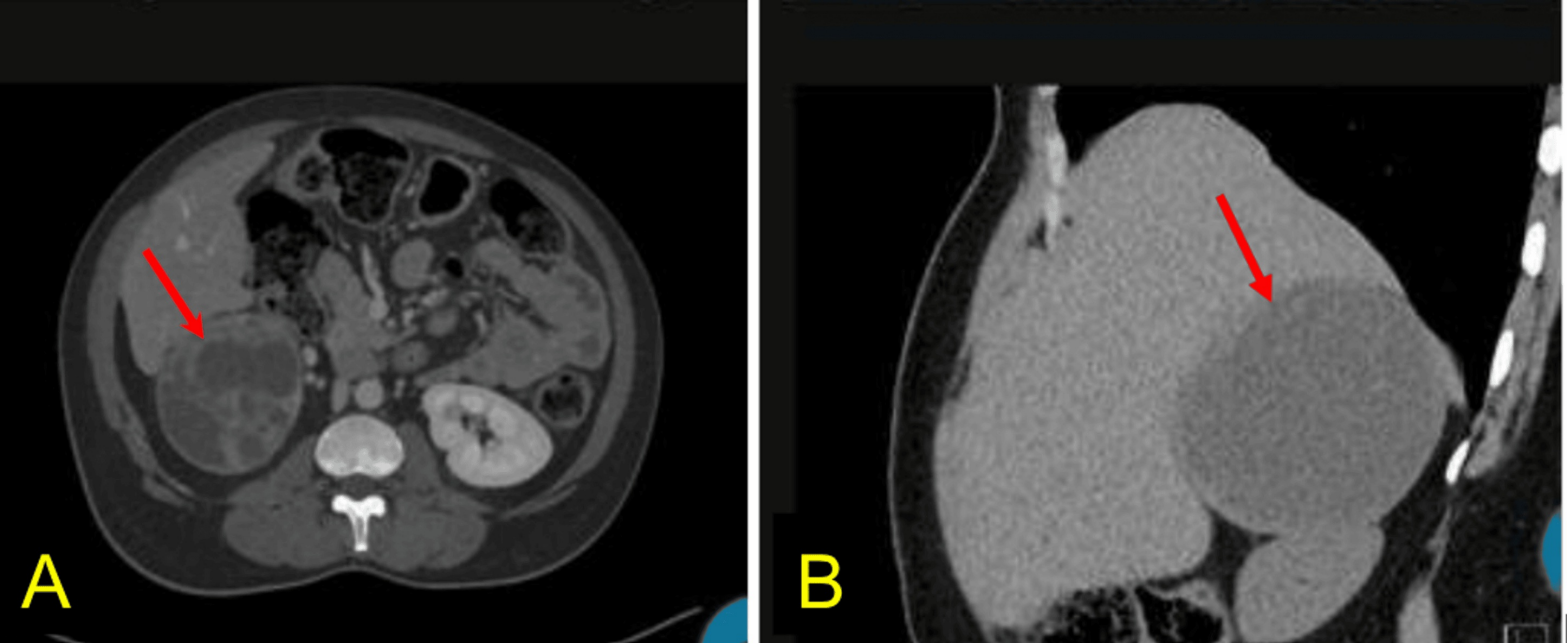
A: CT abdomen with contrast of a sagittal section of a focal soft tissue mass (red arrows) measuring 10.1 x 9.2 x 8.5 cm. The mass demonstrates predominantly low attenuation with internal thin septations without calcification. The margins are smooth, suggesting a non-infiltrative border. There is no capsular retraction or invasion of adjacent structures. B. A cross-sectional CT with contrast showing the lesion (red arrows) with enhancing thin septation, arterial enhancement with increasing enhancement on the venous, and delayed phases with no washout and an internal heterogeneous density with hyperdense foci, consistent with internal hemorrhage. There were no definite signs of vascular invasion or extrahepatic extension.

Ultrasound of the abdomen showed a right adrenal mass measuring 10.2 x 9.4 x 9.0 cm. A decision was made to go for computed tomography (CT) with contrast of the abdomen and pelvis (Figure 1), which showed a large, heterogeneous, septated, well-circumscribed, solid cystic mass with multiple, enhancing thin septations.

Based on these findings, more blood workups were requested. Plasma-free metanephrines were elevated with high normetanephrine and high met-adrenaline. Urine 24-hour metanephrines were markedly increased (Table 2). Eosinophilia and thrombocytosis workup was performed to exclude other reasons, and it was unremarkable, including lactate dehydrogenase (LDH), immunoglobulin E (IgE) level, BCR/ABL (breakpoint cluster region, Abelson murine leukemia), JAK2 tyrosine-protein kinase mutation, ova, and parasite. CT neck, chest, and pelvis were done to rule out metastases, and it was unremarkable.

**Table 2:** Hormonal laboratory tests ACTH: adrenocorticotropic hormone

Test	On admission	Reference range
Urine 24 hrs metanephrines	33.265 mmol/24 hrs	normal <2000 mmol/24 hrs
Plasma Normetadrenaline	18.30 nmol/L	<0.71 nmol/L
Plasma metadrenaline	11.30 nmol/L	<0.36 nmol/L
Plasma 3 ortho-methyl dopamine	0.19 nmol/L	<0.15 nmol/L
ACTH	5.1 pg/ml	<20 pg/ml
Cortisol	337 nmol/L	140-690 nmol/L
Aldosterone (supine)	493 pmol/L	100-450 pmol/L
Renin	>128 mIU/L	4.2 - 60 mIU/L

Blood and urine cultures were unremarkable for any growth, inflammatory markers were negative, and the patient was afebrile and asymptomatic. Therefore, infectious causes of leukocytosis were ruled out, and it was associated with pheochromocytoma. The patient was diagnosed with pheochromocytoma and was initially treated with paracetamol for the headache. The endocrinology team was consulted and advised to target a sitting blood pressure of less than 130/80 mmHg and a standing systolic blood pressure greater than 90 mmHg, and to keep the heart rate between 60 and 80 bpm. The patient was switched from olmesartan to doxazosin since phenoxybenzamine was not available in our hospital. Blood pressure improved, but the patient continued to have a heart rate between 96 and 102 bpm. Therefore, she was started on propranolol at a dosage of 20 mg three times a day. The patient’s symptoms became well-controlled with this medical regimen.

The urology team evaluated the patient and recommended preoperative preparation. The plan was to proceed with interventional radiology embolization followed by laparoscopic adrenalectomy. However, the patient did not return for further evaluation and was subsequently lost to follow-up; therefore, the planned embolization and adrenalectomy were not performed.

## Discussion

Most catecholamine-secreting tumors are sporadic, but they can also occur as a familial disorder. The majority of patients will manifest symptoms and present with typical paroxysmal symptoms, which may include elevated blood pressure, headache, excessive sweating, palpitations, tremors, pallor, shortness of breath, generalized weakness, and symptoms resembling a panic attack [1]. The patient can present with resistant hypertension, which is defined as blood pressure above the goal in a hypertensive patient despite the use of ≥ 3 antihypertensive medications of different classes (one of which should be a diuretic) at optimal or maximally tolerated doses. It also includes patients whose blood pressure is controlled only by the use of ≥ 4 antihypertensive medications [6].

The diagnosis of pheochromocytoma can be confirmed through biochemical testing that measures catecholamine levels in the blood and urine. The next step is to perform imaging studies to identify the size and character of the tumor. CT scan or MRI of the abdomen and pelvis are performed, followed by functional imaging studies in patients with a substantial risk of metastatic disease [4]. Surgical removal is the preferred treatment option. Preoperative embolization in pheochromocytoma is not a routine procedure but may be considered in specific cases such as highly vascular tumors with a risk of excessive intraoperative bleeding. Tumors ≥ 5 cm in pheochromocytoma are an independent risk factor for massive intraoperative blood loss during adrenalectomy; patients with a tumor of ≥ 5 cm are much more likely to experience severe blood loss intraoperatively compared to patients with smaller tumors [7]. The Endocrine Society guidelines emphasize adequate alpha blockade (phenoxybenzamine or doxazosin) followed by beta-blockade if necessary, which is the standard preoperative preparation [8].

We found this case particularly compelling due to the size of the tumor and the associated lab findings. Our patient presented with significant leukocytosis and eosinophilia without any clear infectious causes or any other explanations. A CT scan of the abdomen of our patient is consistent with cystic and hemorrhagic pheochromocytoma, which is less common. Larger pheochromocytomas (>5 cm) have a higher likelihood of internal hemorrhage, which can lead to a cystic appearance on imaging [9].

Studies showed that pheochromocytoma tumors can secrete hormones other than catecholamines, including cytokines, mainly interleukin (IL)-1, IL-6, and tumor necrosis factor alpha (TNF-α) [10,11]. A hypothesis suggested that the association between pheochromocytoma and leukocytosis is due to the overstimulation of β-adrenergic receptors by catecholamines, leading to an increase in white blood cell counts [12]. A case report described a 45-year-old female who was investigated for fever of unknown origin and was found to have leukocytosis. She was eventually diagnosed with pheochromocytoma. It was suggested that the leukocytosis could represent a systemic inflammatory response induced by IL-6 secreted from the pheochromocytoma [13]. Several case reports have described slight and moderate leukocytosis, which showed improvement by controlling blood pressure and removing the tumor mass. Another case report presented a 49-year-old man with uncontrolled hypertension who was diagnosed with pheochromocytoma. His serum investigations showed severe leukocytosis with no other justification. His leukocytosis improved significantly after being started on phenoxybenzamine to prepare for the operation, and it resolved to normal count after the surgery [14].

Another interesting finding in our case is isolated eosinophilia. Eosinophilia workup was done to rule out the common causes, including LDH, IgE, ova and parasite, BCR/ABL mutation, and JAK2 mutation, and they all returned negative. There is limited evidence that indicates a direct link between pheochromocytoma and eosinophilia. In a published case report, a 33-year-old woman who presented with fulminant eosinophilic myocarditis was eventually found to have pheochromocytoma [15]. Studies showed that after evaluating the histopathology of pheochromocytoma of the adrenal medulla, biopsy revealed eosinophilic globules in 63.5% of cases [16]. Although the pathophysiology is not entirely clear, the potential hypothesis behind eosinophilia can be explained by the excessive secretion of catecholamines, IL-5, and other cytokines that are responsible for eosinophil differentiation, activation, and survival [10,11,17].

## Conclusions

Pheochromocytoma can present with a wide range of manifestations beyond its classical triad. In this case, unexplained leukocytosis and marked eosinophilia were notable accompanying findings. Although not established as features of pheochromocytoma yet, such abnormalities may serve as subtle indicators of an underlying catecholamine-secreting tumor. The large cystic adrenal mass with internal hemorrhage also reflects an atypical radiological presentation. Awareness of such unusual clinical and imaging findings can support prompt recognition, timely management, and prevention of pheochromocytomas' potentially life-threatening complications. As this is a single-patient observation, causality cannot be established; however, the findings suggest a possible association between pheochromocytoma and eosinophilia that warrants further research and investigation.
